# Pathway-based gene signatures predicting clinical outcome of lung adenocarcinoma

**DOI:** 10.1038/srep10979

**Published:** 2015-06-04

**Authors:** Ya-Hsuan Chang, Chung-Ming Chen, Hsuan-Yu Chen, Pan-Chyr Yang

**Affiliations:** 1 Institute of Biomedical Engineering, National Taiwan University, No.1, Section 1, Jen-Ai Road, Taipei 100, Taiwan; 2 Institute of Statistical Science, Academia Sinica, 128 Academia Road, Section 2, Nankang, Taipei, Taiwan; 3 Department of Internal Medicine, National Taiwan University College of Medicine, No.1, Section 1, Jen-Ai Road, Taipei, Taiwan

## Abstract

Lung adenocarcinoma is often diagnosed at an advanced stage with poor prognosis. Patients with different clinical outcomes may have similar clinico-pathological characteristics. The results of previous studies for biomarkers for lung adenocarcinoma have generally been inconsistent and limited in clinical application. In this study, we used inverse-variance weighting to combine the hazard ratios for the four datasets and performed pathway analysis to identify prognosis-associated gene signatures. A total of 2,418 genes were found to be significantly associated with overall survival. Of these, a 21-gene signature in the HMGB1/RAGE signalling pathway and a 31-gene signature in the clathrin-coated vesicle cycle pathway were significantly associated with prognosis of lung adenocarcinoma across all four datasets (all p-values < 0.05, log-rank test). We combined the scores for the three pathways to derive a combined pathway-based risk (CPBR) score. Three pathway-based signatures and CPBR score also had more predictive power than single genes. Finally, the CPBR score was validated in two independent cohorts (GSE14814 and GSE13213 in the GEO database) and had significant adjusted hazard ratios 2.72 (p-value < 0.0001) and 1.71 (p-value < 0.0001), respectively. These results could provide a more complete picture of the lung cancer pathogenesis.

Lung cancer, especially non-small cell lung cancer (NSCLC), is the most common cause of cancer-associated mortality worldwide^1^. Adenocarcinoma, a major subtype of NSCLC, is often diagnosed at an advanced stage and generally has a poor clinical outcome^2^ and a relatively poor overall 5-year survival^3^.

The current clinico-pathological staging system is not adequate^4^. Even if lung adenocarcinoma patients have similar clinical characteristics and have tumours at a similar stage, they may experience different clinical outcomes^2^. Due to tumour molecular heterogeneity, some patients will develop metastasis early and some will not^2^. Since high-throughput technology, including microarray and next-generation sequencing, can simultaneously measure the expression of tens of thousands of genes, it can be used to study heterogeneity of the gene expression profile in lung adenocarcinoma^5^,^6^. However, the results of gene expression studies in lung adenocarcinoma have usually been inconsistent due to differences in study design, sample size and analysis strategy^7^. To date, only molecular tests for genomic mutation of *EGFR* and *KRAS* and gene fusion detection of *ALK* have been widely used in clinical practice^8^.

Although previous gene expression microarray studies have shown a statistically significant association of the expression of many individual genes with disease, the findings usually lack biological meaning^9^, which makes it difficult for investigators to interpret their findings^10^. In order to increase the power to detect differentially expressed genes and reduce the difficulty in biological interpretation, gene class-based tests, such as gene set analysis, which combine biological knowledge and gene expression levels, have become widely used^11^. In addition, these methods focus on sets of related genes, rather than on individual genes, as individual functionally associated genes that often show only moderate differential expression can act co-ordinately in the cell, thus magnifying the effect^12^,^13^. From a statistical point of view, this gene class-based method reduces the number of dimensions and increases statistical power, while, from a biological point of view, it should help scientists better understand biological mechanisms within the cell^12^.

Classification of patient risk using a single biomarker that is strongly associated with disease outcome might not be a good strategy, as a dysregulated gene that may not show any obvious association with disease on its own may interact with others in the same pathway, resulting in carcinogenesis or drug resistance^14^,^15^,^16^. It is therefore worth identifying particular sets of genes showing unusual expression that act in the same cancer-associated pathway. In this study, using data from the public gene expression and clinical data on the caArray database of the National Cancer Institute^7^, cancer-associated pathway-based approaches were used to identify pathway-based gene signatures, which may have potential for prognosis prediction and therapeutic target identification in lung adenocarcinoma.

## Results

### Identification of genes with a significant hazard ratio (HR) and their associated pathways

The study population consisted of 443 patients with lung adenocarcinoma from the University of Michigan Cancer Center (UM) (n = 178), the Moffitt Cancer Center (HLM) (n = 79), the Memorial Sloan-Kettering Cancer Center (MSK) (n = 104) and the Dana-Farber Cancer Institute (CAN/DF) (n = 82)^7^. Multivariate Cox proportional hazards regression analysis showed that 2155, 1437, 1164 or 2003 genes in the CAN/DF, HLM, UM or MSK dataset, respectively, were significantly associated with overall survival (data not shown). There were only two genes (*CSNK1A1* and *MYST4*) had significant HRs in all four datasets. After combining the results for the four datasets, a total of 2418 genes showed a significant HR (data not shown). When pathway mapping was used to identify survival-associated biological pathways based on these 2418 genes, 15 pathways in which these genes were enriched were identified (Table 1).

### Pathway-based risk score analysis identifies three pathways that are associated with overall survival in lung adenocarcinoma in all four datasets

To evaluate the impact of signatures in a given pathway on survival, a pathway-based risk score was calculated for this pathway and used, together with survival analysis, to evaluate prognostic ability. Survival analysis showed that three pathways were significantly associated with survival in all four datasets (Table 1). Twenty-one genes significantly associated with survival (11 risk genes and 10 protective genes) were involved in the high-mobility group box 1 (HMGB1)/ receptor for advanced glycation end products (RAGE) signalling pathway (Supplementary Table S2 and Fig. S2 online), 22 (10 risk genes and 12 protective genes) were involved in the beta-adrenergic receptor regulation of the extracellular signal-regulated kinase (ERK) pathway (Supplementary Table S3 and Fig. S3 online) and 31 (11 risk genes and 20 protective genes) were involved in the clathrin-coated vesicle cycle pathway (Supplementary Table S4 and Fig. S4 online).

### Pathway-based signatures of the HMGB1 / RAGE signalling pathway, the beta-adrenergic receptor regulation of the ERK pathway and the clathrin-coated vesicle cycle pathway are significantly associated with clinical outcome of lung adenocarcinoma

**Figure 1:**
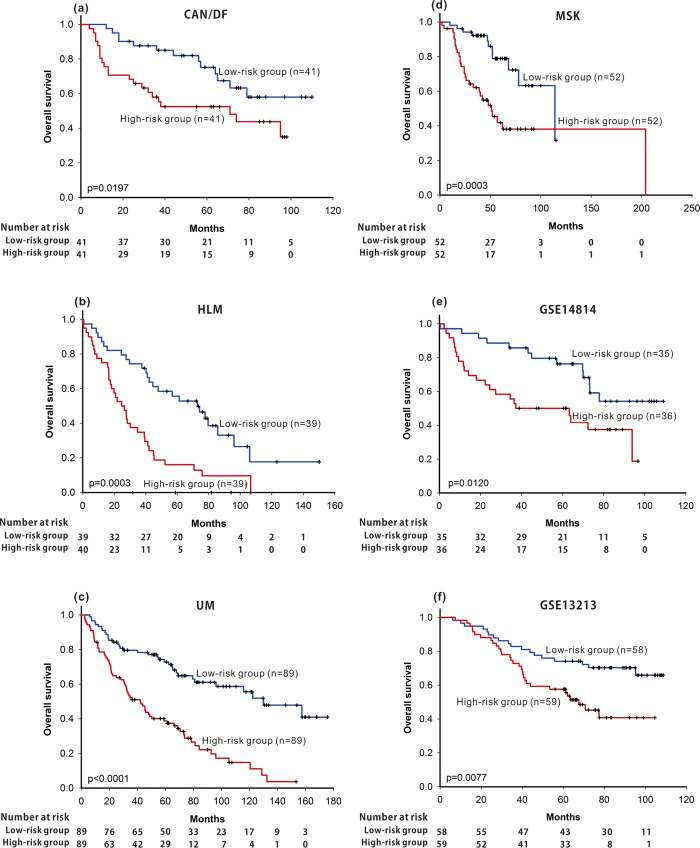
Using HMGB1/RAGE signalling pathway-based signatures as prognosis predictor, Kaplan-Meier survival analysis of patients with lung adenocarcinoma were performed in all four training datasets (CAN/DF, HLM, UK, MSK) and in the two validation cohorts (GSE14814 and GSE1321).

As shown in Fig. 1, using the HMGB1**/**RAGE signalling pathway-based signature containing 21 differentially expressed genes and defining high-risk patients as those with a pathway-based risk score higher than the median, high-risk patients had a significantly shorter median survival than low-risk patients in the CAN/DF dataset (high-risk group 71 months, low-risk group not reached median survival; p = 0.0197), the HLM dataset (high-risk group less than 30 months, low-risk group more than 70 months; p = 0.0003), the UM dataset (high-risk group 42 months, low-risk group 130 months; p < 0.0001) and the MSK dataset (high-risk group 51 months, low-risk group 114 months; p = 0.0003).

**Figure 2:**
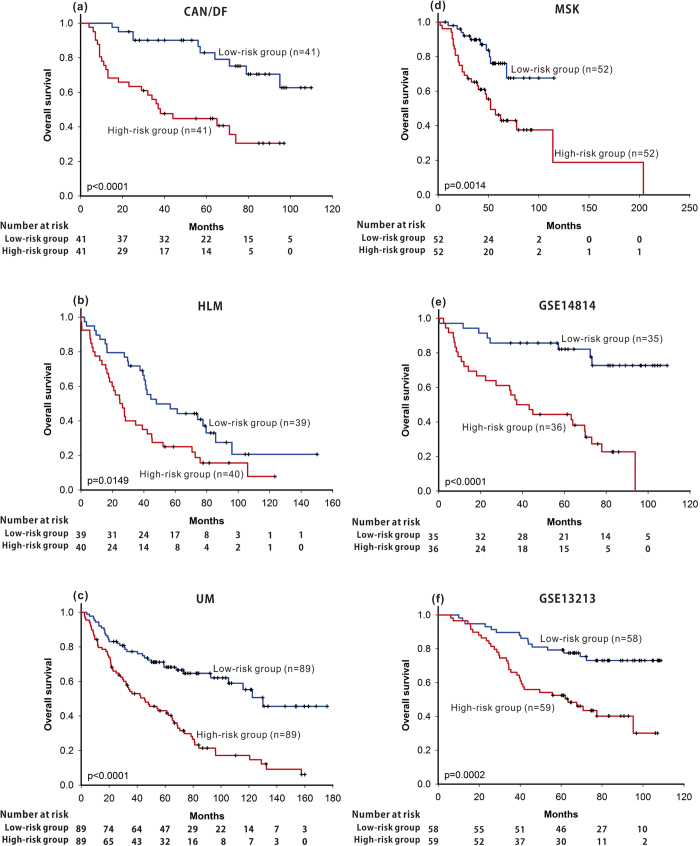
Using pathway-based signatures of beta-adrenergic receptor regulation of the ERK pathway as prognosis predictor, Kaplan-Meier survival analysis of patients with lung adenocarcinoma were performed in all four training datasets (CAN/DF, HLM, UK, and MSK) and in the two validation cohorts (GSE14814 and GSE1321).

**Figure 3:**
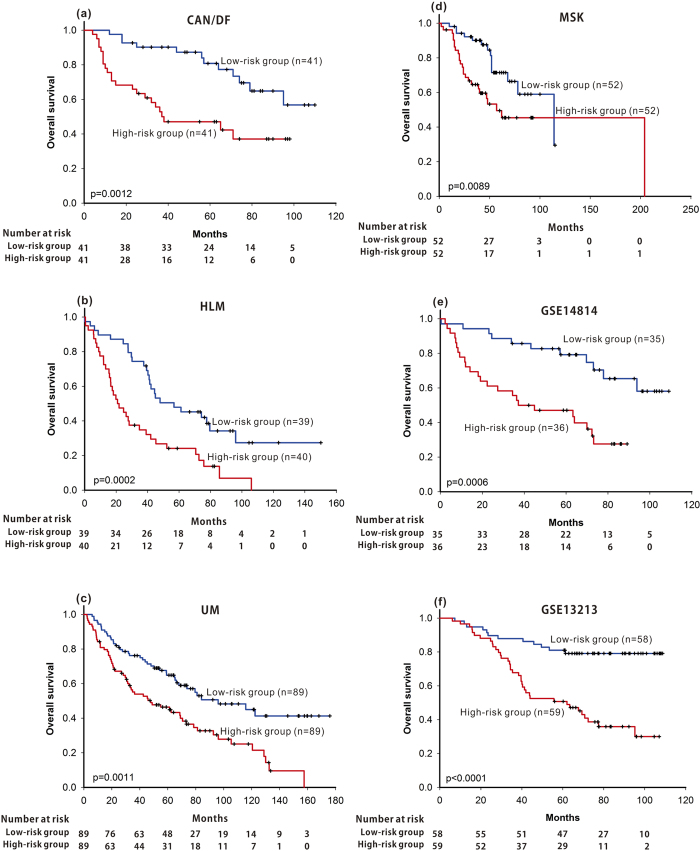
Using pathway-based signatures of clathrin-coated vesicle cycle pathway as prognosis predictor, Kaplan-Meier survival analysis of patients with lung adenocarcinoma were performed in all four training datasets (CAN/DF, HLM, UK, and MSK) and in the two validation cohorts (GSE14814 and GSE1321).

Similar results were obtained using the beta-adrenergic receptor regulation of the ERK pathway-based signature (Fig. 2) or the clathrin-coated vesicle cycle pathway-based signature (Fig. 3). Using the ERK pathway-based signature, the median survival of the high-risk group in the CAN/DF, HLM, UM and MSK datasets was 38, 26, 45 and 52 months, respectively, significantly shorter than that in the low-risk group (not reached median survival, 48 months, 130 months and not reached median survival) (Fig. 2; p < 0.0001, p = 0.0149, p < 0.0001 and p = 0.0014, respectively), while, using the clathrin-coated vesicle cycle pathway-based signature, the median survival of the high-risk groups in the CAN/DF, HLM, UM and MSK datasets were 37, 21, 48 and 57 months, significantly shorter than that of the low-risk group (not reached median survival, 57 months, 96 months and 114 months) (Fig. 3; p = 0.0012, p = 0.0002, p = 0.0011 and p = 0.0089, respectively).

The survival pattern for patients with each of the three pathway-based signatures in the different datasets was interesting. In the CAN/DF dataset, the high-risk group, based, respectively, on the HMGB1 / RAGE signaling pathway, beta-adrenergic receptor regulation of ERK pathway and clathrin-coated vesicle cycle pathway, had a median survival of 71, 38 and 37 months, while the low-risk group did not reach median survival. In the HLM dataset, the high-risk group had a median survival of about 20 months (26, 26 and 21 months), while that of the low-risk group was greater than 45 months (73, 48 and 57 months). In the UM dataset, the median survival for the high-risk group was 42, 45 and 48 months, almost 3 times lower than that in the low-risk group (130, 130 and 96 months). In the MSK dataset, the median survival for the high-risk group was around 50 months (51, 52 and 57 months), while the low-risk group survived longer (114 months, not reach median survival and 114 months).

### A combined pathway-based risk (CPBR) score based on the combined risk scores for the three individual pathways gives a better prediction of clinical outcome in lung adenocarcinoma

**Figure 4:**
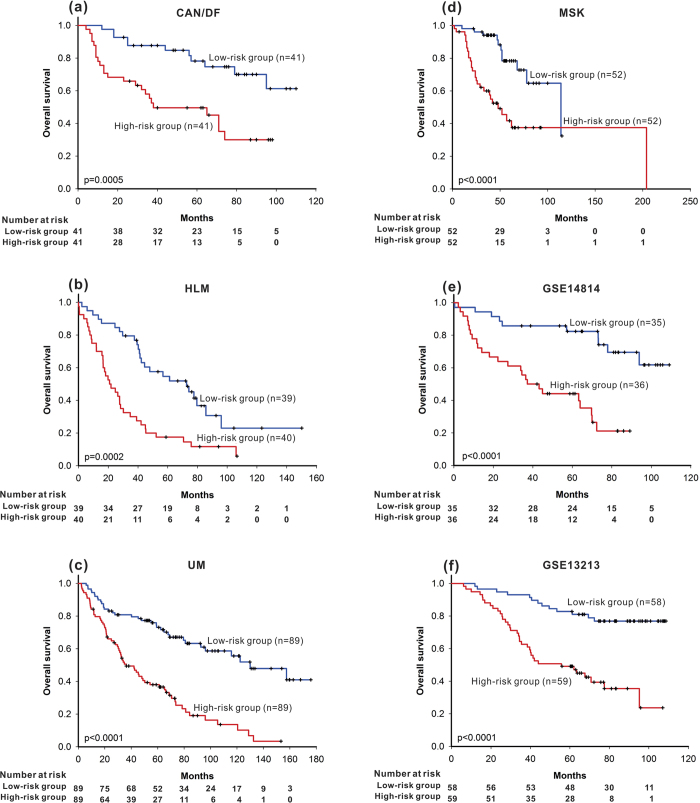
Using combined pathway-based risk (CPBR) score as prognosis predictor, based on the combined risk scores for the three individual pathways, results of survival analysis were shown that overall survival was significantly different between high-risk group and low-risk group in (a) the CAN/DF dataset, (b) the HLM dataset, (c) the UM dataset, (d) the MSK dataset and (e) the validation cohort of GSE14814 (f) the validation cohort of GSE1321, respectively.

To evaluate the prognostic effect of the combined risk scores for these three pathway-based signatures, a CPBR score was computed by linear summation of each of the three pathway-based signature scores multiplied by the weighting coefficient for that pathway obtained by Cox proportional hazards regression. Patients in the high-risk group (risk score higher than the median) had a significantly shorter median survival than those in the low-risk group in all four datasets (CAN/DF p = 0.0005, HLM p = 0.0002, UM p < 0.0001, and MSK p < 0.0001) (Fig. 4a–d).

### Prognostic factors of lung adenocarcinoma

Results of multivariate Cox proportional hazards regression showed that stage effect had significant adjusted HR in each dataset and can be mentioned as an independent prognostic factor (Table 2). After effects of age, sex, and stage were controlled in the multivariate Cox proportional hazards regression model, each pathway-based signature or combined pathway-based risk (CPBR) score was still significant and it was also an independent prognostic factor (Table 2). Particularly, the HRs based on the CPBR score were higher than those obtained using the risk score for any single pathway signature.

### Comparisons of the single biomarker and pathway-based signatures

Multivariate Cox proportional hazards regression was used to examine the predictive power of 2418 genes selected from the inverse-variance weighting method. Numbers of significant genes in four datasets are 820, 574, 429, and 622, respectively. Range of significant HRs was from 0.05 to 11.3 and had high variation between four datasets (Supplementary Table S5). Considering p values and HRs, three pathway-based signatures and CPBR score had more statistical significant than 2418 genes across four datasets (Supplementary Fig. S5). In addition, these pathway-based signatures showed the consistent results in four datasets and portend the better prediction power for prognosis.

### Validation of three pathway-based signatures and the CPBR score in two independent cohorts

Three pathway-based signatures and the CPBR score were validated in two independent cohorts (GSE14814 and GSE13213). Patients with high risk identified from the HMGB1 / RAGE signalling pathway, the beta-adrenergic receptor regulation of the ERK pathway, the clathrin-coated vesicle cycle pathway, or CPBR score had significant shorter overall survival (Fig. 1e, p = 0.0120; Fig. 2e, p < 0.0001; Fig. 3e, p = 0.0006; Fig. 4e, p < 0.0001 ) in the GSE14814 cohort and in the GSE13213 cohort (Fig. 1f, p = 0.0077; Figure2f, p = 0.0002; Fig. 3f, p < 0.0001; Fig. 4f, p < 0.0001), respectively.

In the first validation cohort (GSE14814), age, three pathway-based signatures, and CPBR score had significant HRs. Adjusted HR of CPBR score was 2.72 (95%CI = 1.80 to 4.11 and p < 0.0001). In addition, stage, three pathway-based signatures, and CPBR score had significant HRs in the second validation cohort (GSE13213). Adjusted HR of CPBR score was 1.71(95%CI = 1.45 to 2.03 and p < 0.0001) (Table 3).

## Discussion

In this study, multivariate Cox proportional regression analysis was used to identify potential survival-associated genes in each of the 4 National Cancer Institute datasets, then inverse-variance weighting method was used to combine the results from the four datasets. The use of this method increases the statistical power and provides more robust results^17^. Biological function or pathway analysis was then used to reveal potential biological mechanisms involved in lung cancer, allowing more precise biological interpretation.

In the Cox regression analysis, only two genes, *CSNK1A1* and *MYST4*, were found to be significantly associated with survival in all four datasets. Since the results for the four datasets were not very consistent, we used inverse-variance weighting method to combine the four sets of results to increase statistical power. After pathway analysis, pathway-based risk scores were computed using the level of expression of genes in the same pathway weighted by regression coefficients. Each of the pathway-based scores was found to be a good predictor of clinical outcome. Subjects with a higher pathway-based risk score for a given pathway were classified into the high-risk group based on that pathway. The results showed that 21 of the 65 genes in the HMGB1 / RAGE signalling pathway, 22 of the 70 in the beta-adrenergic receptor regulation of ERK pathway and 31 of the 107 in the clathrin-coated vesicle cycle pathway were significantly associated with clinical outcome of lung cancer. The high-risk group defined using the risk score based on any one of the three individual pathways had a shorter overall survival than the low-risk group.

We then developed a simplified CPBR score to combine the effects of these three signatures, and the results showed that the high-risk group had even shorter overall survival than the low-risk group and that the CPBR score gave a better outcome prediction of outcome of lung adenocarcinoma patients. These results showed that differentially expressed genes in the same pathway might interact with each other and contribute to a worse prognosis.

Some studies have reported that cellular pathway signatures can be useful for treatment development, prognosis prediction and subtype classification in lung cancer^14^,^18^,^19^ and that the identification of important biological pathways containing potential survival-associated genes would help in disease prevention or treatment strategy^16^,^20^. The HMGB1 signalling pathway plays the principal role in the tumorigenesis and progression of many malignant cancers^21^. HMGB1 is a nuclear protein that influences transcription and other nuclear functions and is associated with hallmarks of cancer, including unlimited replication, angiogenesis, apoptosis, self-sufficiency in growth signals, growth inhibitor insensitivity, inflammation, invasion and metastasis^22^,^23^. HMGB1 and its receptor, RAGE, are highly expressed in various malignant tumours, including colorectal and breast cribriform carcinoma^24^. The HMGB1 signalling pathway is also reported to be associated with growth and metastasis of liver cancer and to be a potential therapeutic target for this cancer^21^. In this study, we found that a signature in the HMBG1/RAGE pathway was associated with overall survival, and this might provide insight into the pathogenesis of this cancer. As regards the beta-adrenergic receptor regulation of ERK pathway, psychological distress is a predictor of cancer mortality, especially in lung cancer^25^, and the stress hormone norepinephrine is a potent inducer of migratory activity in lung carcinoma cells and cell migration is mediated by the adrenergic receptor pathway^26^. It has also been reported that beta-blocker therapy can reduce cancer distant metastases, recurrence and mortality rate in breast cancer patients^26^. Clathrin-mediated endocytosis is a regulator of cellular function, and abnormal endocytosis plays a key role in many diseases^27^. The clathrin regulation pathway has been reported to be relevant to Alzheimer’s disease^27^,^28^. These results and our own show that these 3 pathways are important for disease development and that deregulated genes in these pathways might contribute to a worse prognosis. These findings could provide a research direction for further exploration of the mechanism involved in progression of lung adenocarcinoma.

In studies using the same strategy and similar clinical and pathological features and treatment protocol, not all patients with lung cancer show the same clinical outcome and sensitivity to treatment because of the extreme heterogeneity of tumours^2^. Due to the tumor heterogeneity, the multiple genes or pathway based approaches showed the better performance than single marker approach^29^. The development of gene expression profiling should help in demonstrating the heterogeneity of the same tumour type and improve the accuracy of lung cancer risk assessment, clinical prognosis and outcome prediction. Furthermore, it should allow the design of individual targeted therapies for patients^30^.

The limitation of the gene expression profiling or pathway based approach is applications in the clinical practice. The IHC method or FISH method with convenient and low cost of assays is well used in the clinical practice. However, it can be only applied in testing few genes. Several technologies including the multiplexed quantitative reverse-transcriptase polymerase chain reaction (RT-PCR) or digital PCR may provide solutions for the clinical practice of the pathway based signatures. However, it still needs to be evaluated in the future study.

Gene-expression profiling has been used to explore biomarkers associated with subtypes of lung cancer, overall survival and recurrence of cancer^30^,^31^, particular in lung adenocarcinoma^6^,^32^,^33^,^34^. Although signatures associated with lung adenocarcinoma have been reported by several groups, these have tended to be different in different studies; however, the genes in the individual pathways may interact and contribute to cancer pathogenesis or progression^16^,^18^.

Through risk assessment by pathway-based signatures, patients had high risk estimated from pathway based signatures may need to receive different treatments. For example, patients with completely resected stage I NSCLC was recommend for no adjuvant chemotherapy^35^. However, up to 10–20% of above patients will recur or die within 5 years^36^ and may need to receive different clinical treatments. Patients with early recurrence or death are high risk population in the stage I lung cancer and it is still lack of the efficient methods for high risk identification of stage I lung cancer. Hence, the pathway based signature may be benefit to identify high risk group of lung cancer. In addition, pathway based signatures provide systematic point of view for prognosis and the inhibitions of connections between genes within the same pathway may have potential to be therapeutic targets in the future work.

Several limitations of the microarray data are also needed to be considered. First, the high variations of gene expression data were obtained from different microarray platforms due to different probe designs or signal detection methods. Hence, the comparisons between different microarray platforms have to be concerned. Second, the stromal cell contamination varies among dataset and samples, which would give variations in micro array-based analyses.

A potential limitation of this study is that the public datasets used in this study did not provide the information of treatment response and major genomic abnormalities such as *EGFR*, *KRAS*, and *ALK* fusion. It is difficult to estimate the effect of the CPBR in patients with major genomic abnormalities. Additionally, the MetaCore software used enrichment analysis to find the overrepresented pathways and pathways with the smaller size of the gene set might have more chance to reach significance. It is the weakness of the enrichment analysis. Because the false positive may be introduced from the limitation of the MetaCore software, several steps were used to reduce the bias. In the first step, the Bonferroni correction method was applied to reduce potential false positive. There were more than 1000 pathways in the database of the MetaCore. The significant level was corrected from 0.05 to 10^−5^. In the second step, gene-signatures of significant pathways were evaluated in four training datasets. Pathways had significant associations with survival were kept. Finally, candidate pathway-based signatures were validated in two independent cohorts.

Our analysis method of gene expression profiling identified pathway-based signatures closely correlated with clinical outcome of lung adenocarcinoma. We have derived a simple CPBR score which may improve the accuracy of outcome prediction for lung adenocarcinoma, as the CPBR score showed a higher correlation with clinical outcome than gene- or individual pathway-based scores. These results provide a more complete picture of the pathogenesis of lung carcinoma and provide direction for future studies. The CPBR signature may be useful in stratifying subpopulations of lung adenocarcinoma for clinical outcome prediction and individualized therapies for lung adenocarcinoma patients.

## Materials and Methods

### Study population and gene expression data

The study population consisted of 443 patients with lung adenocarcinoma from the University of Michigan Cancer Center (UM) (n = 178), the Moffitt Cancer Center (HLM) (n = 79), the Memorial Sloan-Kettering Cancer Center (MSK) (n = 104) and the Dana-Farber Cancer Institute (CAN/DF) (n = 82)^7^. The clinical characteristics of all subjects are briefly summarized in Supplementary Table S1 online. In all 4 studies, gene expression profiles were measured using Affymetrix HG-U133A microarrays and the same experimental protocols were used. The gene expression and clinical data for these 4 groups were obtained from https://array.nci.nih.gov/caarray/project/details.action? project.id= 182.

An additional published dataset (n = 71) from the University of Toronto (accession number GSE14814 in the GEO database, http://www.ncbi.nlm.nih.gov/geo/query/acc.cgi?acc=GSE14814) and dataset (n = 117) from the Nagoya University in Japan (accession number GSE13213 in the GEO database, http://www.ncbi.nlm.nih.gov/geo/query/acc.cgi?acc=GSE%2013213) were used to validate our findings.

### Data preprocessing

The intensity values for gene expression in the four datasets were preprocessed independently to eliminate experimental noise before data analysis. To reduce variation among microarrays in a given data set, the intensity values for each sample were normalized using the quantile-normalized method^37^. Finally, each intensity value underwent base 2 logarithm transformation. The flowchart for the analysis is shown in Supplementary Fig. S1 online.

### Identification of genes showing statistically significant differential expression using multivariate Cox proportional hazards regression

In order to determine whether genes were significantly associated with overall survival, multivariate Cox proportional hazards regression using the clinical covariates of age, sex and stage was performed on each of the 4 datasets. Each gene in a given dataset was assigned a hazard ratio (HR), and the corresponding p value was estimated by Cox regression analysis, giving 4 HR and 4 p values for each gene, then the inverse-variance weighting method was used to combine the HRs from the four datasets^38^. The advantages of combining the results from the different datasets were an increase in statistical power and the identification of more robust cancer signatures^17^. If the confidence interval for the combined HR for a given gene did not overlap with that for the baseline risk (HR = 1), the gene was considered a potential marker that was significantly associated with survival. The HRs were then used to evaluate correlations between overall survival and the level of expression of genes; if the HR for a given gene was >1, it was defined as a potential risk gene, if not, it was defined as a potential protective gene.

### Pathway analysis and risk score calculation

Genes that were differentially expressed and associated with survival were further analysed for biological function or involvement in different pathways using pathway maps of the MetaCore^TM^ version 6.13 (Thomson Reuters, New York, NY). Because more than 1000 pathways are included in the MetaCore database, the Bonferroni correction was used to avoid multiple testing issues. For each of the 15 pathways found to contain differentially expressed genes, a pathway-based risk score was calculated for each subject in each data set to determine the impact of the pathway on the prognosis. The risk score was a linear combination of the values for the expression of each gene in the pathway multiplied by a weighting value for each gene estimated using Cox proportional hazards regression. Using this approach, we found signatures in three pathways that were significantly associated with survival in lung adenocarcinoma. A combined pathway-based risk (CPBR) score was then derived by linear combination of the pathway-based risk score for each of the three pathways multiplied by a weighting value for that pathway estimated using Cox proportional hazards regression. We then verified this CPBR score using an independent cohort.

### Survival analysis

In the pathway-based study, the pathway-based median risk score was taken as the cut-off point for high or low-risk group classification, while, in the CPBR score study, the median of the CPBR score was used as the cut-off. The Kaplan-Meier method was used to generate survival curves and the difference between survival curves was evaluated using the log-rank test. All tests were two-tailed, and p values less than 0.05 were considered to be significant.

### CPBR score validated in another independent cohort

Both survival analysis and calculation method of pathway-based scores for three selected pathways and the CPBR score were the same as previous section described. HR of the CPBR score was estimated from multivariate Cox proportional hazards regression with the clinical covariates of age, sex, stage, and treatment method after operation.

## Additional Information

**How to cite this article**: Chang, Y.-H. *et al*. Pathway-based gene signatures predicting clinical outcome of lung adenocarcinoma. *Sci. Rep*. **5**, 10979; doi: 10.1038/srep10979 (2015).

## Supplementary Material

Supplementary Information

## Figures and Tables

**Table 1:** **Possible clinical outcome-related pathways in lung adenocarcinoma.**

Pathway	P value	Number ofsignificant genes (total gene number)	Significantly associated with survival in all four datasets	Significantly associated with survival in three datasets	Significantly associated with survival in two datasets
Immune response_HMGB1/RAGE signalling pathway	9.73E-08	21 (65)	V		
Development_Beta-adrenergic receptor regulation of ERK	8.92E-06	22(70)	V		
Transport_Clathrin-coated vesicle cycle	4.81E-06	31(107)	V		
Transport_RAN regulation pathway	2.44E-06	16(47)		V	
Cell cycle_Role of Nek in cell cycle regulation	1.34E-07	19(61)		V	
Development_Glucocorticoid receptor signalling	8.63E-06	12(41)		V	
Immune response_IL-6 signalling pathway	6.25E-07	13(33)		V	
Development_WNT signalling pathway. Part 2	2.56E-06	19(84)		V	
Cell cycle_Chromosome condensation in prometaphase	7.34E-10	14(33)			V
Cell cycle_Role of APC in cell cycle regulation	1.23E-11	27(54)			V
Development_WNT signalling pathway. Part 1. Degradation of beta-catenin in the absence of WNT signalling					
	4.67E-06	11(29)			V
Cell cycle_Spindle assembly and chromosome separation	3.06E-09	29(94)			V
Cell cycle_Transition and termination of DNA replication	1.27E-07	20(37)			V
Cell cycle_Start of DNA replication in early S phase	1.01E-06	16(43)			V
Cell cycle_The metaphase checkpoint	5.48E-06	14(36)			V

The symbol “V” denotes that this pathway-based risk score was significantly associated with survival.

**Table 2:** Adjusted hazard ratios of the different pathway-based signatures in four training datasets.

Variable	CAN/DF		HLM		UM		MSK					
	HR (95% CI)^a^	P value^a^	HR (95% CI) a	P value^a^	HR (95% CI) a	P value^a^	HR (95% CI) a	P value a				
**HMGB1/RAGE signalling pathway**												
Gene signature	1.31(1.15-1.49)	<0.0001	1.60(1.35-1.90)	<0.0001	1.59(1.36-1.85)	<0.0001	1.52(1.29-1.78)	<0.0001				
Age	1.07(1.03-1.12)	0.0016	1.03(1.00-1.06)	0.0622	1.02(1.00-1.04)	0.1319	1.00(0.96-1.04)	0.9490				
Sex	1.20(0.59-2.47)	0.6111	0.58(0.33-1.01)	0.0537	1.66(1.07-2.57)	0.0224	1.41(0.71-2.82)	0.3280				
**Stage**												
I	1.00		1.00		1.00		1.00					
II	2.64(1.33-5.25)	0.0056	2.86(1.45-5.63)	0.0024	2.26(1.37-3.72)	0.0014	2.49(0.96-6.44)	0.0610				
III			5.19(2.57-10.49)	<0.0001	4.28(2.61-7.01)	<0.0001	6.55(2.92-14.68)	<0.00001				
**Beta-adrenergic receptor regulation of ERK pathway**												
Gene signature	1.37(1.21-1.56)	<0.0001	1.46(1.27-1.68)	<0.0001	1.62(1.32-2.00)	<0.0001	1.36(1.19-1.56)	<0.0001				
Age	1.11(1.05-1.17)	0.0001	1.03(1.00-1.06)	0.0394	1.02(1.00-1.04)	0.1153	1.00(0.97-1.05)	0.7060				
Sex	1.10(0.54-2.26)	0.7858	0.76(0.45-1.28)	0.3028	1.64(1.06-2.53)	0.0263	1.07(0.53-2.13)	0.8560				
**Stage**												
I	1.00		1.00		1.00		1.00					
II	2.05(1.03-4.10)	0.0418	4.50(2.19-9.25)	<0.0001	2.62(1.58-4.36)	0.0002	2.07(0.81-5.32)	0.1300				
III			7.73(3.73-16.02)	<0.0001	4.30(2.62-7.03)	<0.0001	5.65(2.56-12.46)	<0.0001				
**Clathrin-coated vesicle cycle pathway**												
Gene signature	1.18(1.11-1.26)	<0.0001	1.18(1.10-1.27)	<0.0001	1.35(1.19-1.54)	<0.0001	1.23(1.12-1.34)	<0.0001				
Age	1.09(1.04-1.14)	0.0006	1.03(1.00-1.06)	0.0670	1.01(0.99-1.03)	0.2585	1.00(0.96-1.04)	0.9640				
Sex	1.20(0.58-2.49)	0.6320	0.85(0.50-1.43)	0.5421	1.71(1.11-2.63)	0.96(0.47-1.95)	0.0148	0.9010				
**Stage**												
I	1.00		1.00		1.00		1.00					
II	2.88(1.45-5.74)	0.0026	3.05(1.55-6.00)	0.0012	2.00(1.21-3.30)		1.59(0.62-4.08)	0.3320				
III			5.60(2.80-11.22)	<0.0001	4.64(2.83-7.61)		6.40(2.86-14.35)	<0.0001				
**Combined pathway-based risk (CPBR)**												
**score**	2.72(1.89-3.90)	<0.0001	2.72(1.93-3.83)	<0.0001	2.72(1.98-3.74)	<0.0001	2.72(1.88-3.93)	<0.0001				
Age	1.10(1.04-1.15)	0.0003	1.03(1.00-1.06)	0.0782	1.02(0.99-1.04)	0.1509	1.00(0.96-1.04)	0.8940				
Sex	1.17(0.57-2.43)	0.6674	0.59(0.34-1.02)	0.0569	1.69(1.10-2.62)	0.0178	1.23(0.61-2.47)	0.5520				
Stage												
I	1.00		1.00		1.00		1.00					
II	2.54(1.28-5.04)	0.0079	3.78(1.87-7.63)	0.0002	2.38(1.44-3.93)	0.0007	2.17(0.84-5.61)	0.1090				
III			6.80(3.31-13.98)	<0.0001	4.29(2.61-7.05)	<0.0001	6.67(2.94-15.16)	<0.0001				

HR: hazard ratio; 95% CI: 95% confidence interval.

^a^Adjusted by covariates.

**Table 3:** Adjusted hazard ratios of the different pathway-based signatures in two validation cohorts.

	GSE14814		GSE13213	
Variable	HR (95% CI) a	P value^a^	HR (95% CI) ^a^	P value^a^
**HMGB1/RAGE signalling pathway-based risk score**				
Gene Signature	1.64(1.24-2.18)	0.0005	2.56(1.65-3.97)	<0.0001
Age	1.08(1.02-1.13)	0.0033	1.01(0.98-1.03)	0.6500
Sex	2.12(1.00-4.49)	0.0503	1.30(0.71-2.38)	0.3910
Stage				
I	1.00		1.00	
II	1.61(0.78-3.30)	0.1950	1.83(0.74-4.55)	0.1920
III			4.75(2.51-8.97)	<0.0001
**Beta-adrenergic receptor regulation of ERK pathway**				
Gene Signature	2.21(1.53-3.17)	<0.0001	1.70(1.39-2.07)	<0.0001
Age	1.07(1.01-1.12)	0.014	1.01(0.98-1.04)	0.3964
Sex	1.62(0.76-3.44)	0.2080	1.80(0.97-3.33)	0.0603
Stage				
I	1.00		1.00	
II	1.36(0.66-2.81)	0.4080	1.73(0.70-4.26)	0.2371
III			4.92(2.58-9.38)	<0.0001
**Clathrin-coated vesicle cycle pathway**				
Gene Signature	1.49(1.21-1.84)	0.0002	1.56(1.33-1.84)	<0.0001
Age	1.07(1.02-1.13)	0.0094	1.01(0.98-1.04)	0.5290
Sex	2.18(1.01-4.71)	0.0468	1.25(0.68-2.30)	0.4730
Stage				
I	1.00		1.00	
II	1.18(0.56-2.50)	0.6571	1.06(0.42-2.67)	0.8960
III			3.60(1.92-6.76)	<0.0001
**Combined pathway-based risk (CPBR)**				
Gene Signature	2.72(1.80-4.11)	<0.0001	1.71(1.45-2.03)	<0.0001
Age	1.07(1.01-1.13)	0.0163	1.01(0.98-1.04)	0.4970
Sex	1.81(0.85-3.84)	0.1225	1.41(0.76-2.61)	0.2820
Stage				
I	1.00		1.00	
II	1.35(0.64-2.85)	0.4323	1.21(0.47-3.14)	0.6920
III			5.04(2.65-9.55)	<0.0001

HR: hazard ratio; 95% CI: 95% confidence interval.

^a^Adjusted by covariates.
