# Supplementary material for: Pathway-based gene signatures predicting clinical outcome of lung adenocarcinoma
Source: Sci Rep. 2015 Jun 4;5:10979. doi: 10.1038/srep10979 (PMC4455286; doi:10.1038/srep10979)
Supplement: Supplementary Information [file srep10979-s1.pdf]

Pathway-based gene signatures predicting clinical outcome of lung adenocarcinoma

Ya-Hsuan Chang[1], Chung-Ming Chen[1], Hsuan-Yu Chen[2]*, and Pan-Chyr Yang[3]

[1]Institute of Biomedical Engineering, National Taiwan University, No1, Sec. 1, Jen - Ai Rd., Taipei 100, Taiwan

[2]Institute of Statistical Science, Academia Sinica, 128 Academia Road, Section 2, Nankang, Taipei, Taiwan

[3]Department of Internal Medicine, National Taiwan University College of Medicine, 1Jen-Ai Road, Section 1, Taipei, Taiwan

*Corresponding Author: Hsuan-Yu Chen

Phone: +(886)-2-6614-5675

Fax: +(886)-2-2782-1523

Email: hychen@stat.sinica.edu.tw

Address: 128 Academia Road, Section 2, Nankang, Taipei 11529, Taiwan

Table S1. Clinical characteristics of 443 lung adenocarcinoma in 4 institutites.

	CAN/DF	HLM	UM	MSK
Sample size	82	79	178	104
Age(mean, s.d.)	62(10)	67(10)	64(10)	65(10)
Gender(% male)	56%	51%	57%	36%
Stage I	68%	54%	65%	61%
Stage II	32%	26%	17%	19%
Stage III	0%	19%	18%	20%
Median follow-up(months)	51	39	53	43
Number of deaths	35	60	103	39

Table S2. Hazard ratios estimated from meta-analysis of genes in the HMGB1/RAGE signaling pathway.

Probesets	Gene	Uni gene	HR[a]	95% C.I.[a]	
204314_s_at	CREB1	Hs.584750	0.70	0.52	0.94
209190_s_at	DIAPH1	Hs.529451	0.61	0.46	0.82
213514_s_at	DIAPH1	Hs.529451	0.73	0.58	0.92
204363_at	F3	Hs.62192	1.25	1.10	1.42
214938_x_at	HMGB1	Hs.434102	0.58	0.37	0.89
216244_at	IL1RN	Hs.81134	1.15	1.03	1.27
205207_at	IL6	Hs.654458	1.18	1.02	1.35
202670_at	MAP2K1	Hs.145442	1.36	1.03	1.79
213490_s_at	MAP2K2	Hs.465627	1.33	1.03	1.71
205698_s_at	MAP2K6	Hs.463978	1.32	1.08	1.61
211500_at	MAPK11	Hs.57732	1.29	1.03	1.60
206106_at	MAPK12	Hs.432642	1.21	1.02	1.42
212535_at	MEF2A	Hs.268675	0.69	0.51	0.93
209199_s_at	MEF2C	Hs.699175	0.78	0.65	0.94
209200_at	MEF2C	Hs.699175	0.84	0.75	0.94
211524_at	NFKB2	Hs.73090	1.13	1.00	1.28
214062_x_at	NFKBIB	Hs.9731	0.86	0.76	0.97
212239_at	PIK3R1	Hs.132225	0.76	0.60	0.97
212240_s_at	PIK3R1	Hs.132225	0.77	0.61	0.97
207821_s_at	PTK2	Hs.395482	0.75	0.58	0.96
208820_at	PTK2	Hs.395482	0.73	0.57	0.92
201087_at	PXN	Hs.446336	1.50	1.05	2.14
206036_s_at	REL	Hs.631886	0.71	0.57	0.89
203843_at	RPS6KA3	Hs.445387	0.76	0.59	0.97
204035_at	SCG2	Hs.516726	1.13	1.02	1.26

HR: hazard ratio; 95%C.I.: 95% confidence interval

[a] Adjusted by age, sex, and stage

Table S3. Hazard ratios estimated from meta-analysis of genes in the Beta-adrenergic receptors regulation of ERK pathway.

Probesets	Gene	Uni gene	HR[a]	95% C.I. [a]	
209321_s_at	ADCY3	Hs.705457	1.43	1.12	1.84
204497_at	ADCY9	Hs.391860	0.77	0.68	0.88
204498_s_at	ADCY9	Hs.391860	0.69	0.55	0.87
206170_at	ADRB2	Hs.591251	0.84	0.70	1.00
217523_at	CD44	Hs.502328	0.86	0.75	0.98
201179_s_at	GNAI3	Hs.73799	1.64	1.20	2.25
211858_x_at	GNAS	Hs.125898	1.28	1.00	1.65
200852_x_at	GNB2	Hs.185172	1.50	1.11	2.03
211871_x_at	GNB5	Hs.155090	1.35	1.01	1.81
212294_at	GNG12	Hs.431101	0.77	0.61	0.96
205184_at	GNG4	Hs.159711	1.24	1.06	1.44
207157_s_at	GNG5	Hs.645427	1.70	1.16	2.49
206896_s_at	GNG7	Hs.515544	0.79	0.64	0.98
202670_at	MAP2K1	Hs.145442	1.36	1.03	1.79
213490_s_at	MAP2K2	Hs.465627	1.33	1.03	1.71
204524_at	PDPK1	Hs.459691	0.70	0.53	0.93
32029_at	PDPK1	Hs.459691	0.69	0.50	0.96
206370_at	PIK3CG	Hs.32942	0.86	0.78	0.96
205111_s_at	PLCE1	Hs.655033	0.85	0.72	1.00
208652_at	PPP2CA	Hs.483408	0.67	0.47	0.97
200605_s_at	PRKAR1A	Hs.280342	0.74	0.56	0.97
214487_s_at	RAP2A	Hs.508480	1.29	1.03	1.62
215992_s_at	RAPGEF2	Hs.113912	0.83	0.71	0.98
205590_at	RASGRP1	Hs.591127	0.84	0.73	0.97

HR: hazard ratio; 95%C.I.: 95% confidence interval

[a] Adjusted by age, sex, and stage

Table S4. Hazard ratios estimated from meta-analysis of genes in the Clathrin-coated vesicle cycle pathway.

Probesets	Gene	Uni gene	HR[a]	95% C.I. [a]	
215787_at	ACTA2	Hs.500483	0.77	0.62	0.95
205423_at	AP1B1	Hs.368794	1.33	1.01	1.75
205196_s_at	AP1S1	Hs.563509	1.22	1.02	1.44
211779_x_at	AP2A2	Hs.19121	0.67	0.48	0.93
212159_x_at	AP2A2	Hs.19121	0.67	0.48	0.92
212161_at	AP2A2	Hs.19121	0.68	0.47	0.97
202120_x_at	AP2S1	Hs.119591	1.66	1.18	2.33
211047_x_at	AP2S1	Hs.119591	1.39	1.04	1.86
208750_s_at	ARF1	Hs.286221	1.29	1.01	1.66
201358_s_at	COPB1	Hs.339278	0.57	0.37	0.88
201264_at	COPE	Hs.10326	1.46	1.12	1.89
217726_at	COPZ1	Hs.505652	1.48	1.06	2.05
201279_s_at	DAB2	Hs.481980	0.81	0.67	0.99
201280_s_at	DAB2	Hs.481980	0.82	0.70	0.95
210757_x_at	DAB2	Hs.481980	0.80	0.65	0.99
217886_at	EPS15	Hs.83722	0.67	0.48	0.95
217887_s_at	EPS15	Hs.83722	0.77	0.59	1.00
205425_at	HIP1	Hs.329266	0.79	0.66	0.95
215074_at	MYO1B	Hs.439620	0.73	0.65	0.82
212511_at	PICALM	Hs.163893	0.70	0.52	0.93
217861_s_at	PREB	Hs.279784	1.49	1.07	2.07
200863_s_at	RAB11A	Hs.321541	1.42	1.07	1.89
219681_s_at	RAB11FIP1	Hs.696035	0.79	0.67	0.94
211961_s_at	RAB7A	Hs.15738	0.62	0.41	0.93
203150_at	RABEPK	Hs.19012	1.34	1.01	1.80
201542_at	SAR1A	Hs.499960	0.72	0.52	0.98
218254_s_at	SAR1B	Hs.432984	0.72	0.57	0.90
201583_s_at	SEC23B	Hs.369373	1.36	1.07	1.72
212900_at	SEC24A	Hs.595540	0.68	0.51	0.91
212902_at	SEC24A	Hs.595540	0.72	0.56	0.93
215209_at	SEC24D	Hs.189641	0.76	0.66	0.88
215641_at	SEC24D	Hs.189641	0.73	0.63	0.85
209091_s_at	SH3GLB1	Hs.136309	1.44	1.11	1.89
210101_x_at	SH3GLB1	Hs.136309	1.42	1.06	1.91
212111_at	STX12	Hs.523855	0.66	0.49	0.89

212112_s_at	STX12	Hs.523855	0.63	0.45	0.90
212799_at	STX6	Hs.518417	0.60	0.42	0.86
203457_at	STX7	Hs.593148	0.72	0.52	1.00
212631_at	STX7	Hs.593148	0.75	0.58	0.95
212632_at	STX7	Hs.593148	0.63	0.45	0.88
213480_at	VAMP4	Hs.6651	0.66	0.47	0.93
202546_at	VAMP8	Hs.534373	0.74	0.59	0.92
207967_at	VPS45	Hs.443750	0.78	0.63	0.96

HR: hazard ratio; 95%C.I.: 95% confidence interval

[a] Adjusted by age, sex, and stage

Table S5. Results of multivariate Cox proportional hazards regression for 2418 genes, selected by the inverse-variance weighting method for four datasets.

Dataset	Number of significant hazard ratio	Minimum significant hazard ratio	Maximum significant hazard ratio
CAN/DF	820	0.05	8.35
HLM	574	0.11	4.65
UM	429	0.40	3.36
MSK	622	0.05	11.3

Figure legends

Figure S1. The flowchart of data analysis.

Figure S2. Immune response_HMGB1/RAGE signaling pathway.

Figure S3. Development_Beta-adrenergic receptors regulation of ERK pathway.

Figure S4. Transport_Clatherin-coated vesicle cycle pahway.

Figure S5. Distributions of hazard ratios and p values, estimated from multivariate Cox proportional hazards regression. Black dots denoted 2418 genes and red dots denoted three pathway-based signatures and CPBR score

Figure S1

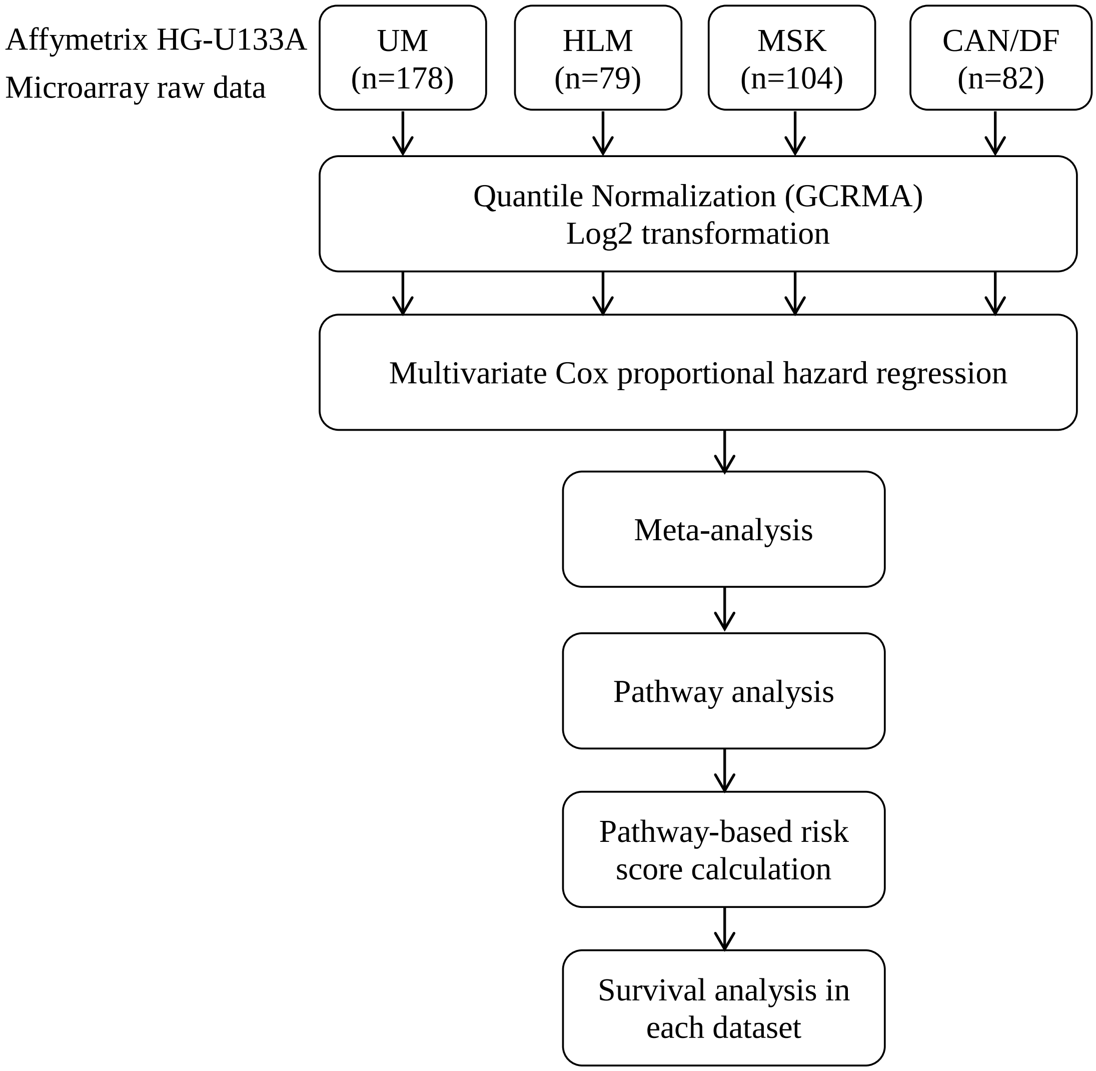
Affymetrix HG-U133A
Microarray raw data
UM (n=178)
HLM (n=79)
MSK (n=104)
CAN/DF (n=82)
Quantile Normalization (GCRMA)
Log2 transformation
Multivariate Cox proportional hazard regression
Meta-analysis
Pathway analysis
Pathway-based risk score calculation
Survival analysis in each dataset

Figure S2.

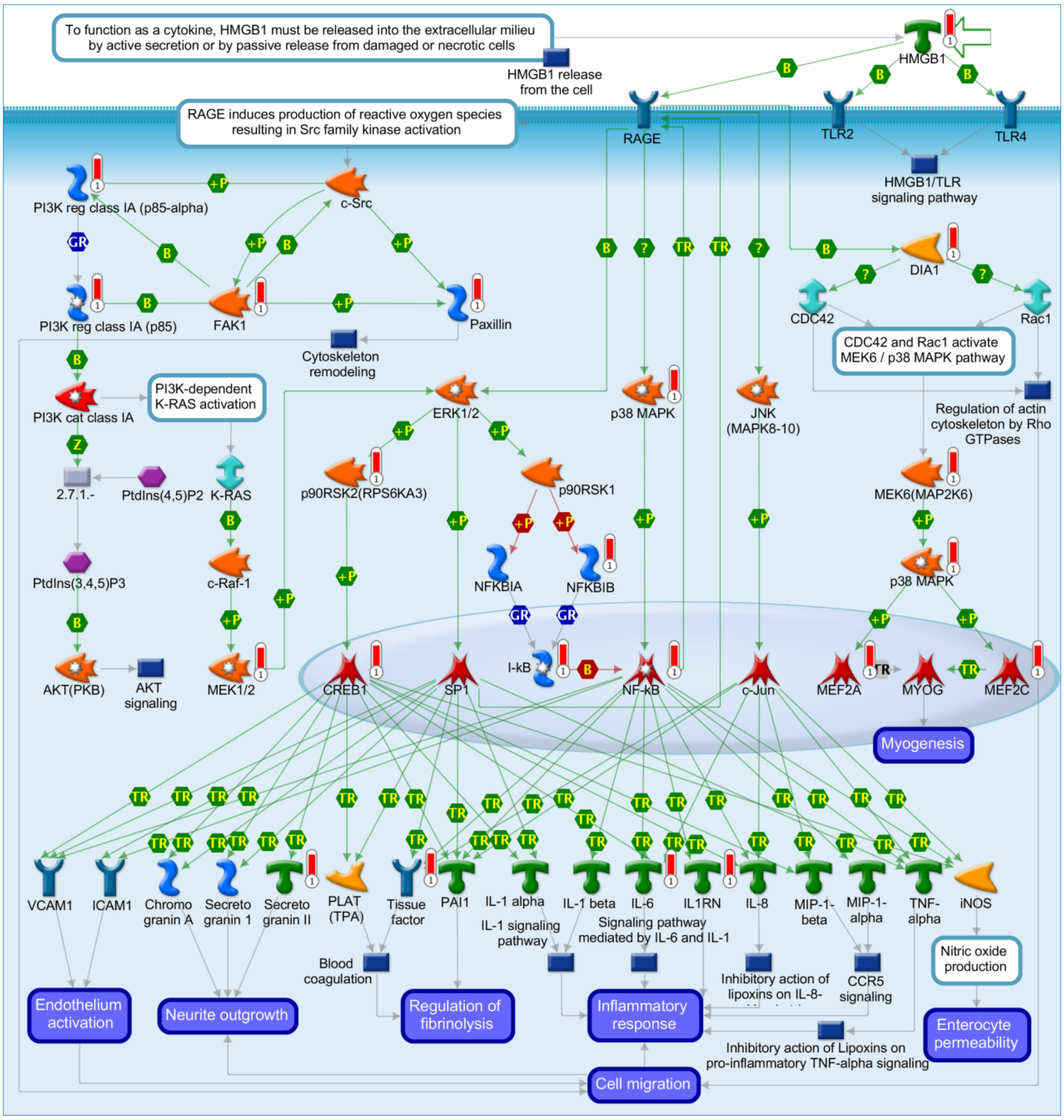

To function as a cytokine, HMGB1 must be released into the extracellular milieu by active secretion or by passive release from damaged or necrotic cells
HMGB1 release from the cell
HMGB1
RAGE
TLR2
TLR4
HMGB1/TLR signaling pathway
RAGE induces production of reactive oxygen species resulting in Src family kinase activation
PI3K reg class IA (p85-alpha)
c-Src
PI3K reg class IA (p85)
FAK1
Paxillin
Cytoskeleton remodeling
DIA1
CDC42
Rac1
CDC42 and Rac1 activate MEK6 / p38 MAPK pathway
PI3K cat class IA
PI3K-dependent K-RAS activation
ERK1/2
p38 MAPK
JNK (MAPK8-10)
Regulation of actin cytoskeleton by Rho GTPases
2.7.1.-
PtdIns(4,5)P2
K-RAS
p90RSK2(RPS6KA3)
p90RSK1
MEK6(MAP2K6)
PtdIns(3,4,5)P3
c-Raf-1
NFKBIA
NFKBIB
p38 MAPK
AKT(PKB)
AKT signaling
MEK1/2
CREB1
SP1
I-kB
NF-kB
c-Jun
MEF2A
MYOG
MEF2C
Myogenesis
VCAM1
ICAM1
Chromo granin A
Secreto granin 1
Secreto granin II
PLAT (TPA)
Tissue factor
PAI1
IL-1 alpha
IL-1 beta
IL-6
IL1RN
IL-8
MIP-1-beta
MIP-1-alpha
TNF-alpha
iNOS
IL-1 signaling pathway
Signaling pathway mediated by IL-6 and IL-1
Nitric oxide production
Blood coagulation
Inhibitory action of lipoxins on IL-8-
CCR5 signaling
Endothelium activation
Neurite outgrowth
Regulation of fibrinolysis
Inflammatory response
Enterocyte permeability
Inhibitory action of Lipoxins on pro-inflammatory TNF-alpha signaling
Cell migration

Figure S3.

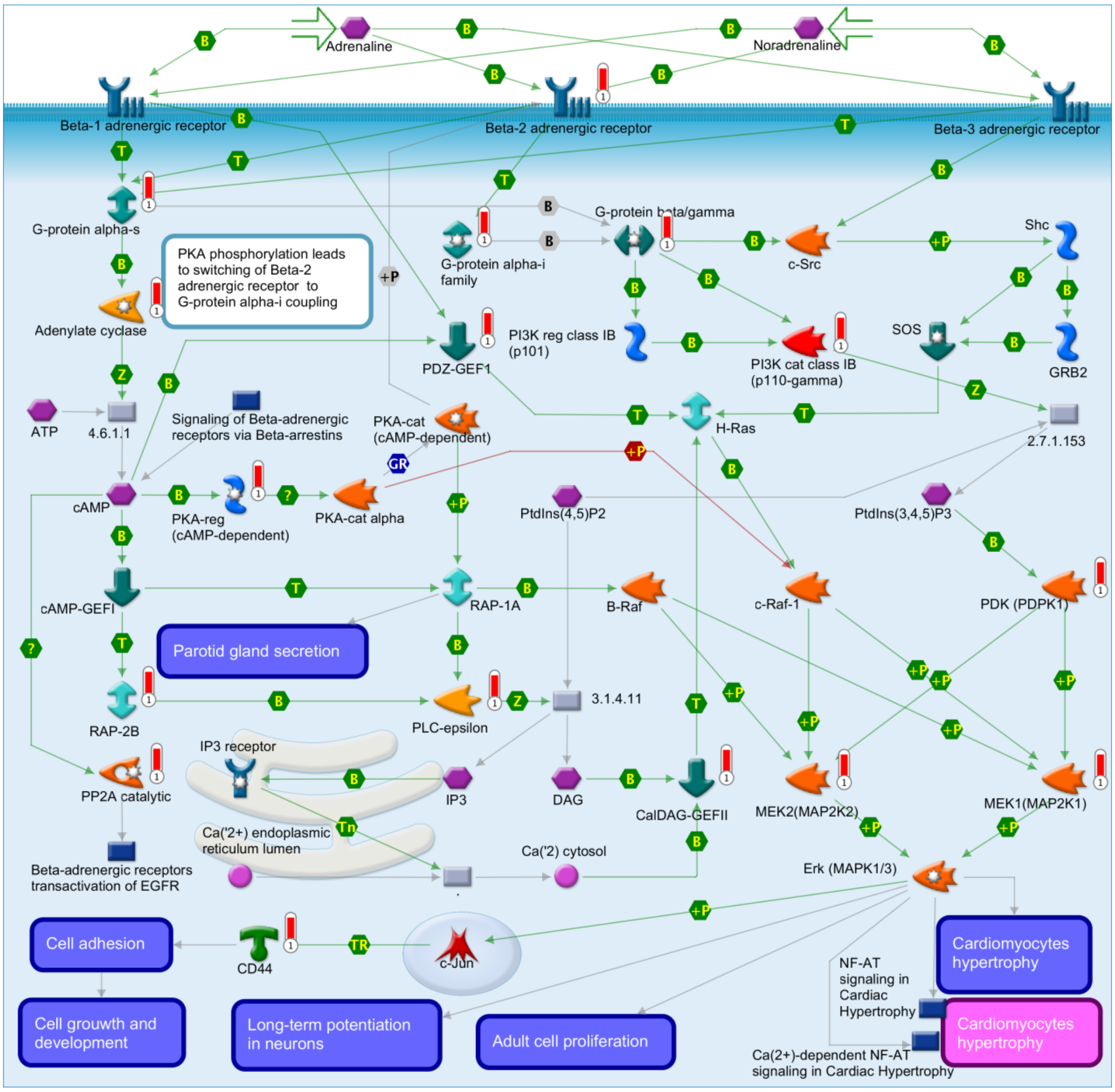

Adrenaline
Noradrenaline
Beta-1 adrenergic receptor
Beta-2 adrenergic receptor
Beta-3 adrenergic receptor
G-protein alpha-s
G-protein alpha-i family
G-protein beta/gamma
c-Src
Shc
PKA phosphorylation leads to switching of Beta-2 adrenergic receptor to G-protein alpha-i coupling
Adenylate cyclase
PDZ-GEF1
PI3K reg class IB (p101)
PI3K cat class IB (p110-gamma)
SOS
GRB2
ATP
4.6.1.1
Signaling of Beta-adrenergic receptors via Beta-arrestins
PKA-cat (cAMP-dependent)
H-Ras
2.7.1.153
cAMP
PKA-reg (cAMP-dependent)
PKA-cat alpha
PtdIns(4,5)P2
PtdIns(3,4,5)P3
cAMP-GEFI
RAP-1A
B-Raf
c-Raf-1
PDK (PDPK1)
Parotid gland secretion
RAP-2B
PLC-epsilon
3.1.4.11
IP3 receptor
PP2A catalytic
IP3
DAG
CalDAG-GEFII
MEK2(MAP2K2)
MEK1(MAP2K1)
Ca('2+) endoplasmic reticulum lumen
Ca('2) cytosol
Erk (MAPK1/3)
Beta-adrenergic receptors transactivation of EGFR
Cell adhesion
CD44
c-Jun
Cardiomyocytes hypertrophy
NF-AT signaling in Cardiac Hypertrophy
Cell growuth and development
Long-term potentiation in neurons
Adult cell proliferation
Cardiomyocytes hypertrophy
Ca(2+)-dependent NF-AT signaling in Cardiac Hypertrophy

Figure S4.

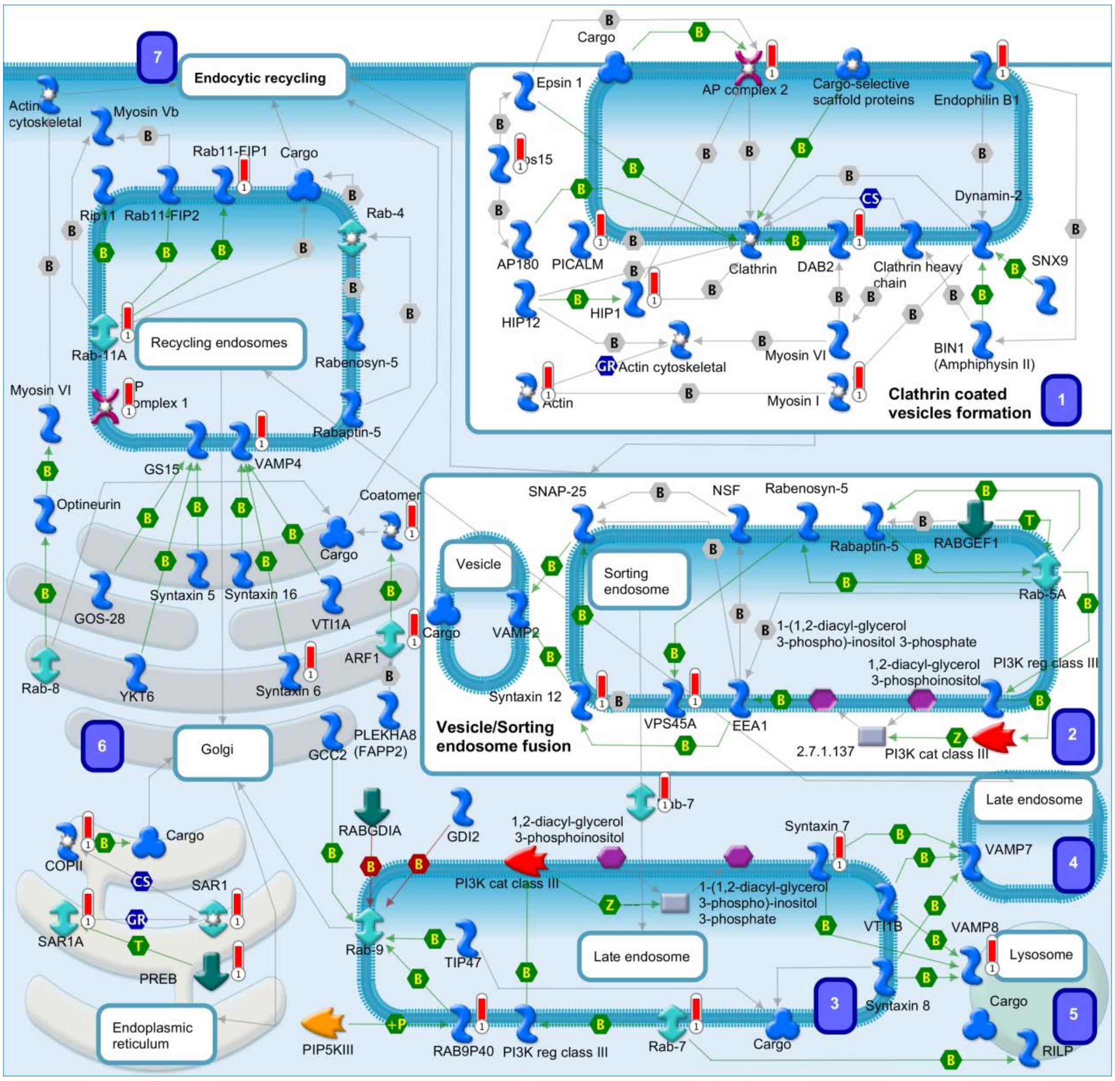
Endocytic recycling
7
Actin cytoskeletal
Myosin Vb
Rab11-FIP1
Cargo
Rip11
Rab11-FIP2
Rab-4
Rab-11A
Recycling endosomes
Rabenosyn-5
Myosin VI
Rabaptin-5
GS15
VAMP4
Optineurin
Coatomer
Cargo
Syntaxin 5
Syntaxin 16
GOS-28
VTI1A
ARF1
Rab-8
YKT6
Syntaxin 6
6
Golgi
GCC2
PLEKHA8 (FAPP2)
Cargo
COPII
SAR1
SAR1A
PREB
Endoplasmic reticulum
Cargo
Epsin 1
AP complex 2
Cargo-selective scaffold proteins
Endophilin B1
Dynamin-2
AP180
PICALM
Clathrin
DAB2
Clathrin heavy chain
SNX9
HIP12
HIP1
Actin cytoskeletal
Myosin VI
BIN1 (Amphiphysin II)
Actin
Myosin I
Clathrin coated vesicles formation
1
SNAP-25
NSF
Rabenosyn-5
Rabaptin-5
RABGEF1
Vesicle
Sorting endosome
Rab-5A
Cargo
VAMP2
1-(1,2-diacyl-glycerol 3-phospho)-inositol 3-phosphate
1,2-diacyl-glycerol 3-phosphoinositol
PI3K reg class III
Syntaxin 12
VPS45A
EEA1
Vesicle/Sorting endosome fusion
2.7.1.137
PI3K cat class III
2
Late endosome
RABGDIA
GDI2
1,2-diacyl-glycerol 3-phosphoinositol
Syntaxin 7
VAMP7
4
PI3K cat class III
1-(1,2-diacyl-glycerol 3-phospho)-inositol 3-phosphate
VTI1B
VAMP8
Rab-9
TIP47
Late endosome
Lysosome
Syntaxin 8
Cargo
3
5
PIP5KIII
RAB9P40
PI3K reg class III
Rab-7
Cargo
RILP

Figure S5.

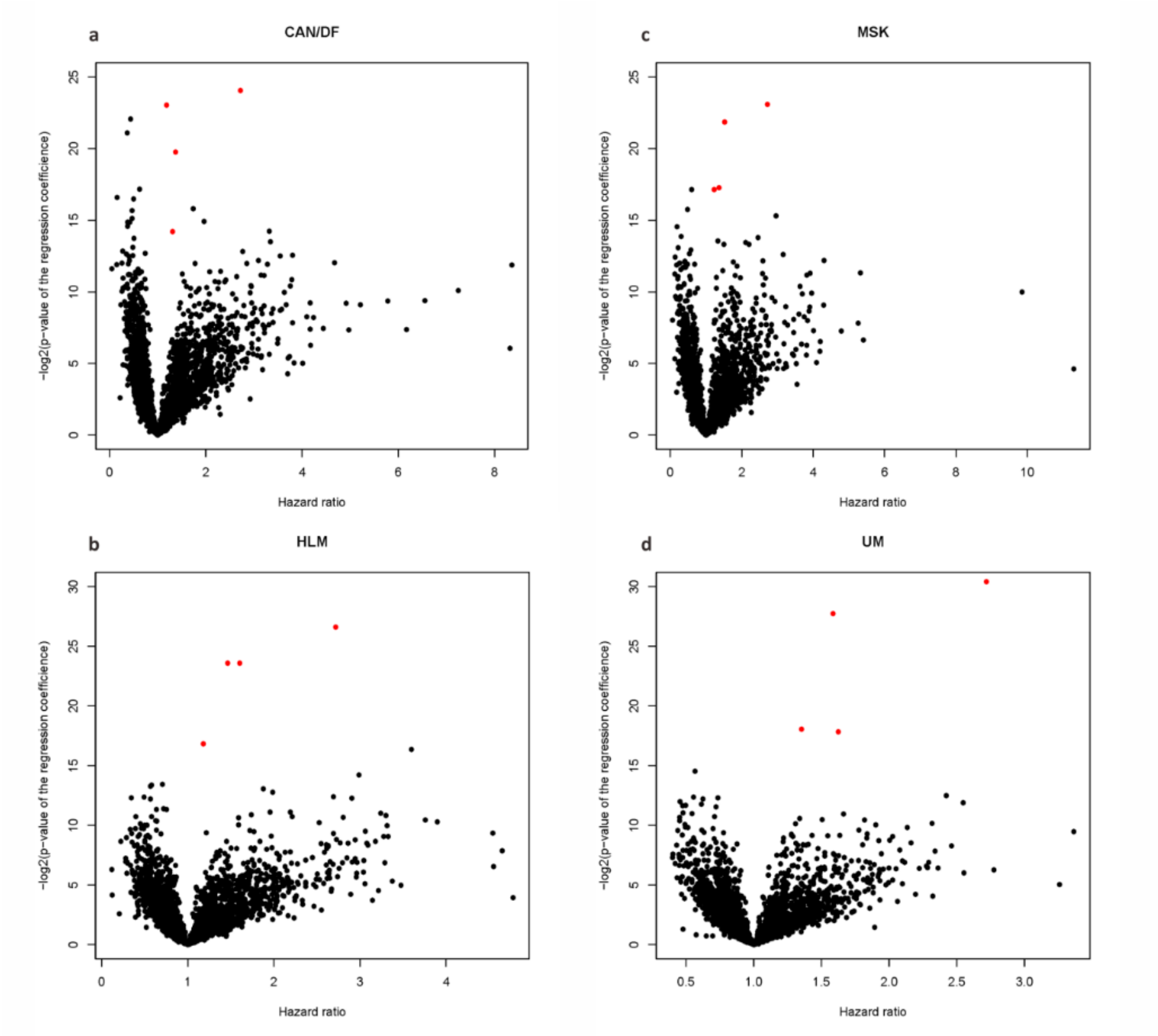

a
CAN/DF
−log2(p−value of the regression coefficience)
Hazard ratio
c
MSK
−log2(p−value of the regression coefficience)
Hazard ratio
b
HLM
−log2(p−value of the regression coefficience)
Hazard ratio
d
UM
−log2(p−value of the regression coefficience)
Hazard ratio
